# Synthesis and Analysis of Polymorphic Silver Nanoparticles and Their Incorporation into the Polymer Matrix

**DOI:** 10.3390/polym14132666

**Published:** 2022-06-30

**Authors:** Oksana Velgosova, Livia Mačák, Maksym Lisnichuk, Marek Vojtko

**Affiliations:** 1 Institute of Materials and Quality Engineering, Faculty of Materials Metallurgy and Recycling, Technical University of Kosice, Letná 9/A, 042 00 Kosice, Slovakia; livia.macak@tuke.sk; 2 Faculty of Science, Institute of Physics, Pavol Jozef Šafárik University in Kosice, Park Angelinum 9, 040 01 Kosice, Slovakia; maksym.lisnichuk@upjs.sk; 3 Division of Ceramic and Non-Metallic Systems, Institute of Materials Research, Slovak Academy of Sciences, Watsonova 47, 040 01 Kosice, Slovakia; mvojtko@saske.sk

**Keywords:** silver nanoparticles, chemical synthesis, TEM, optical properties

## Abstract

A chemical method was successfully used to synthesize silver nanoparticles (AgNPs) with various shapes. The shape of the nanoparticles affects the color of the colloid (spherical—yellow solution, triangular—blue, a mixture of spherical and triangular—green). The NaBH_4_, which acts as the main reducing agent and H_2_O_2_ have a significant impact on the shape of AgNPs. It has also been shown that the ratio between precursor, reducing, and the stabilizing agent is crucial for the formation of the required nanoparticles. The light sensitivity of AgNPs and the presence of H_2_O_2_ lead to a significant change in AgNPs’ shape and size with time and to the formation of the dichroic effect. UV–vis spectrophotometry, TEM, SEM/FIB, and EDX methods were used to analyze the shape, size, and composition of the nanoparticles. Polymer matrix composite with AgNPs was prepared by the “ex-situ” method.

## 1. Introduction

The chemical methods of nanoparticles synthesis belong to the most used. Among all chemical methods, direct chemical reduction is widely used due to its relative simplicity, high yield, low financial costs, and the possibility to synthesize differently shaped nanoparticles simply by changing a concentration of reduction agents [1,2]. It should be noted that the use of toxic chemicals, which are necessary for the synthesis of AgNPs, is a disadvantage of this method and can cause environmental pollution if handled incorrectly. However, in the production of specific shapes of nanoparticles, the chemical method is irreplaceable.

The chemical reduction method can be used for the synthesis of different types of metal nanoparticles (Ag, Au, Cu, Fe, etc.). Among all the metal nanoparticles, silver has a very important position, not only because of its physical (conductivity), and chemical (catalysis) properties, but also because silver nanoparticles have remarkable antibacterial properties. Moreover, colloids of silver nanoparticles are able to show a remarkable optical property.

Silver nanoparticles and silver colloids have wide use as diagnostic or therapeutic agents in nanomedicine, AgNPs have good catalytic properties, they can be used as antibacterial and disinfection agents; their optical properties are used for scientific applications and for art (as a coloring agent) [3,4]. Silver colloids offer different colors depending on the size and shape of the nanoparticles; in addition, under certain conditions, colloidal silver solutions can be dichroic. In optics, a dichroic material is a material that causes visible light to be split up into distinct beams of different wavelengths (colors). In the case of dichroic silver colloid, the color changes depending on the direction of light transmission. In reflected light, the solution can show, for instance, a green color, whereas when viewed in transmitted light, the solution appears yellow [5,6].

There are only several scientific papers that deal with the preparation and use of colloids of silver and gold nanoparticles with various shapes and even fewer works that deal with the dichroic phenomenon. The preparation of specific shapes of the nanoparticles, especially dichroic nanoparticles, is relatively complicated, as this phenomenon depends on the presence of highly polymorphic nanoparticles. Much effort has been made to understand the mechanism, and many hypotheses have been proposed to explain the formation of such highly anisotropic structures [7,8,9].

The chemical method of the synthesis of silver nanoparticles requires several chemicals, each of which plays a specific role (reducing, stabilization, capping). During the synthesis, chemicals interact with precursor and with each other, not insignificant is the impact of the freshness of prepared solutions and conditions of the synthesis process (stirring speed and the temperature of reactants). Therefore, it is not easy to describe exactly the process of synthesis, besides the reduction of Ag^+^, degradation or dissolution of synthesized nanoparticles can also occur, and silver ions are released back into the solution [7]. In the early stages of research, the most popular theory of nanoparticles growth was the face-blocking theory but later it was proved that the crystal symmetry of the starting nuclei is also very important [9,10].

The stability of nanoparticles is another important factor. It is well known that silver nanoparticles are sensitive to light, solution pH, and reaction reagents. This sensitivity can cause changes in shape and size or cause the degradation of nanoparticles over time. Ensuring the stability of AgNP is not an easy task. One possibility is the incorporation of nanoparticles into the polymer matrix, which can ensure the stability of the nanoparticles and increase the application possibilities of the nanoparticle colloids. There are many polymer matrices suitable for preparing polymer matrix composites doped by the metal nanoparticles, and poly (vinyl alcohol) (PVA) is one of the most used. PVA is a water-soluble synthetic polymer. PVA is used in various medical applications due to its biocompatibility, low tendency for protein adhesion, and low toxicity. Specific uses include cartilage replacements, contact lenses, and eye drops. The combination of PVA with silver nanoparticles can change the properties of PVA, as silver has attractive physical, chemical, and toxic properties. PVA-AgNPs composites can be used as thin films that provide antibacterial properties, fibers prepared by electrospinning can be used as filters, PVA-AgNPs nonwovens can be prepared for use in medicine (masks, protective suits) or in the textile industry, the optical properties of AgNPs can be used in optics for preparing dichroic or interference filters, etc.

Understanding the contribution of individual reactants is crucial for the successful preparation of AgNPs colloids with specific properties. Since even small changes in the synthesis process (e.g., the sequence of reactants, mixing rate, or synthesis temperature) can easily affect the formation of nanoparticles, it is necessary to define the exact process conditions. The aim of the work was to prepare various-shaped AgNPs by chemical method, analyze the influence of reagents on the nanoparticles shape and size, and monitor the stability of nanoparticles. Prepared nanoparticles were incorporated into the polymer matrix by the ex-situ method, and the impact of AgNPs on the optical properties of polymer composites was analyzed.

## 2. Materials and Methods

The chemical synthesis was used to produce silver nanoparticles (AgNPs). As a silver precursor, silver nitrate (>98%) purchased from Mikrochem Ltd., Pezinok, Slovakia was used. Sodium borohydride (≥98%), sodium citrate (TSC) (≥99%), hydrogen peroxide (30%), and polyvinylpyrrolidone (PVP) were also purchased from Mikrochem Ltd., Pezinok, Slovakia, and used as received. De-ionized water was used for preparing all solutions.

The amount and concentrations of reagents are given in Table 1, four AgNPs colloids (with different concentrations of NaBH_4_) were prepared. The procedure for AgNPs colloids preparation was as follows:

1. An advance prepared AgNO_3_ solution was vigorously mixed without heating for 5 min on a magnetic stirrer (the weight concentration of Ag in 0.11 mM solution is 12 mg/L);
2. Then, sodium citrate (TSC), PVP, and hydrogen peroxide were added in this order;
3. After 5 min of stirring, the required amounts of ice-cold, freshly-prepared sodium borohydride were added;
4. Solutions were stirred until the color change occurred (10–20 min);
5. Prepared solutions were stored at room temperature in daylight;
6. The ex-situ method of PVA-AgNPs composite preparation:
  ⚬ Chosen colloidal solutions were centrifuged (9000 rpm for 20 min) to concentrate the nanoparticles (100 mL of AgNPs colloidal solutions was concentrated to 15 mL);
  ⚬ Concentrated nanoparticles have been added to the polyvinyl alcohol matrix, an 8% solution of PVA-AgNPs was prepared. PVA-AgNPs solution was stirred at 70 °C for several hours to secure good distribution of AgNPs in matrix;
  ⚬ Subsequently, still-hot solutions of PVA-AgNPs composite were used to prepare the thin layers, the mixture was poured onto the substrate, and spread to a thickness of 0.3 mm; prepared thin layers were allowed to dry at ambient temperature.

The AgNPs were monitored by UV-vis Spectrometer (UNICAM UV-vis Spectrophotometer UV4, Waltham, MA, USA). The size and morphology of the nanoparticles were studied by means of TEM (JEOL model JEM-2000FX, an accelerating voltage of 200 kV, Tokyo, Japan). The image analysis (ImageJ software, free product) was used for the analysis of Ag nanoparticles’ size distribution. The morphology of AgNPs was observed by the scanning electron microscopy SEM/FIB (SEM/FIB ZEISS-AURIGA Compact, Zeiss, Oberkochen, Germany). The phase composition of the samples was analyzed using *X*-ray diffraction analysis (XRD, ZEISS, Oberkochen, Germany).

## 3. Results and Discussion

### 3.1. Synthesis Process

The easy and time-saving chemical method was used to prepare AgNPs colloids by reaction of silver nitrate (a precursor of silver ions), sodium citrate (a stabilization agent with a weak reducing effect, TSC), sodium borohydride (strong reduction agent), hydrogen peroxide, and polyvinylpyrrolidone (to control the shape formation) [11,12,13].

During the synthesis of silver nanoparticles, a lot of reactions take place and each one has an important role [7,14,15]. The first one is the reaction between silver nitrate and sodium citrate. As sodium citrate is a weak reduction agent, which gives rise to nuclei/seeds, the other reagents must be added for the complete reduction and stabilization of nanoparticles. The latest research on citrate impact shows that citrate ions alone can stabilize the silver nanoplates by stabilizing formed silver nuclei through preferential binding to the (111) facets [7]. However, its stabilization effect must be supported by another ligand. The combination of citrate and PVP helps to stabilize and control the formation of nanoparticles shape. PVP is utilized as a dispersant; PVP provides covalent organic ligands and allows steric repulsion to prevent AgNPs aggregation; moreover, it can protect AgNPs surfaces from oxidation and instability [16]. Thiele et al. [17] analyzed the influence of PVP content on nanoparticle synthesis and have proved that a low concentration of PVP could insure well-dispersed silver nanoparticles. It was originally claimed that TSC and PVP have a crucial role in determining the shape of nanoparticles, but the latest research results show that H_2_O_2_ plays an irreplicable role in shape formation too [7].

The impact of H_2_O_2_ on silver ions strongly depends on the pH of the solution [15,18,19]. H_2_O_2_ can act as a reducing agent under alkaline conditions [18,19]. It was proved that despite the reducing ability, the concentration of H_2_O_2_ cannot drastically affect the reduction rate of the Ag^+^ [15], but under certain conditions, H_2_O_2_ is able to change the shape of already synthesized nanoparticles by etching [7,14,20]. The experiments made at different pH of the solution and with a different volume ratio of H_2_O_2_ [14,21] show that when Ag nanoparticles and H_2_O_2_ are mixed under neutral conditions, H_2_O_2_ acts as an oxidizing agent and the surface of Ag nanoparticles can be dissolved. It was also confirmed that the sole use of H_2_O_2_, without stabilizing substances, caused the production of large particles on the micron scale. To produce nanometre-scale particles, the PVP and NaBH_4_ presence have a great influence. NaBH_4_ is used as a strong reducing agent. It is well-known that strong reducing agents have high nucleation ability and favor the formation of many small nanoparticles.

It can be concluded that nanoparticles of various shapes and sizes can be prepared by appropriate selection of the amount and the ratio of individual reactants. However, the size of nanoparticles and their stability are most affected by the concentrations of stabilizing agents and silver precursor, and by the pH of the solution [15]. At appropriate conditions, H_2_O_2_, as a powerful oxidant, favors the production of silver nanoplates by inducing the formation of planar twinned defects and removing other less stable structures. However, by harnessing the oxidative power of H_2_O_2_, planar (triangular) AgNPs can be converted to truncated triangles or irregularly shaped nanoparticles, which can lead to optical changes in the solution.

### 3.2. UV-Vis Spectroscopy

**Figure 1:**
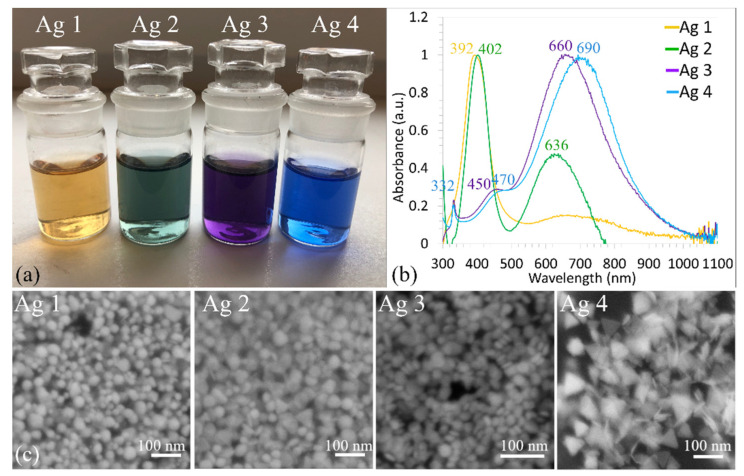
Color of AgNPs colloids immediately after preparation (**a**); UV-vis spectra of AgNPs colloids day 0 (**b**); and SEM images of sample Ag 1, 2, 3 and 4 (**c**).

After the vigorous mixing of all reagents, a visible color change was observed Figure 1a. In general, this is the first evidence of successful nanoparticle synthesis. In the case of metallic nanoparticles, such as AgNPs, the free movement of electrons between the valance band and conduction bands is possible due to the very narrow gap between them. The electrons on the surface of AgNPs produce the local surface plasmon resonance (LSPR), which is a resonant oscillation of conduction electrons in response to the incident light [22]. Due to the LSPR, various colors of AgNPs colloids can be observed. The colloids’ colors depend upon various factors, e.g., size, the shape of AgNPs, and the surrounding medium [23].

The sample Ag 1 (with the smallest amount of NaBH_4_ (0.45 mL)) turned yellow after the synthesis of nanoparticles. The samples Ag 2, Ag 3, and Ag 4 where 0.84, 1, and 1.5 mL of NaBH_4_ were added turned green, violet, and blue, respectively Figure 1a. It is obvious that the various quantity of NaBH_4_ influenced the synthesis process and based on the colors of the solutions we can suggest that different shape of nanoparticles was synthesized.

The best and most popular analytical method to analyze colloidal solutions is UV–vis spectrophotometry. The normalized UV–vis spectra of prepared AgNPs colloids are in Figure 1b. The different plasmonic properties of the solutions indicate the nanoparticles of various shapes and/or sizes [24].

Colloid Ag 1 showed a strong SPR band with good symmetry and the absorption peak wavelength (*λ*_max_) at 392 nm was observed Figure 1b. The results of several authors [2,7,14,15,16] confirmed that if ABS_max_ is around 390–400 nm and the SPR band shows one strong peak, then mainly small, spherical nanoparticles in a narrow size range are present. The narrower the peak width, the smaller the standard deviation of the particle diameter or aspect ratio, respectively. Based on the presence, shape, and position of one strong peak Figure 1b, it can be concluded that the synthesized nanoparticles (sample Ag 1) are small, spherical, and in a narrow size distribution interval. This assumption confirmed the SEM image of sample Ag 1 Figure 1c.

UV-vis absorption spectrum of the Ag 2 colloid showed two peaks Figure 1b, the first strong, and slim peak at *λ*_max_ = 402 nm, and the second at a smaller wavelength of 636 nm. The presence of two strong peaks indicates the presence of two different shaped nanoparticles, as is confirmed in Figure 1c where spherical and triangular nanoparticles can be observed.

A significant shift to longer wavelength was observed in the case of Ag 3 and 4 colloids; *λ*_max_ was ~660, and ~690 nm, respectively. Both SPR bands are very similar in shape (Figure 1b); the presence of three peaks can be observed in both SPR bands, two of them are strong and sharp (*λ*_max_ 330 nm and 660–900 nm) and one is more like a weak shoulder peak, approximately at 450–480 nm. Based on the presence of more peaks, it can be assumed that various sizes and/or shapes of AgNPs were formed. A lot of authors studied chemical methods of AgNPs synthesis and observed the formation of three peaks (at ~330,470, and ~700 nm) [12,21,24]. They conclude that the peak at the high wavelength (higher than 510 nm) indicates the presence of triangular nanoprism structures and the color of solutions is in shades of blue-violet [12,13,24,25]. The other two bands correspond to the in-plane dipole plasmon and out-of-plane quadrupole resonance [13,25]. Based on this, it is possible to predict that mainly triangular-shaped nanoparticles have formed and, together with triangular ones, it is possible to expect a certain number of, e.g., spherical nanoparticles [15,24]. This assumption was confirmed by SEM images where spherical and disclike nanoparticles Figure 1c and triangular Figure 1c, were observed.

**Figure 2:**
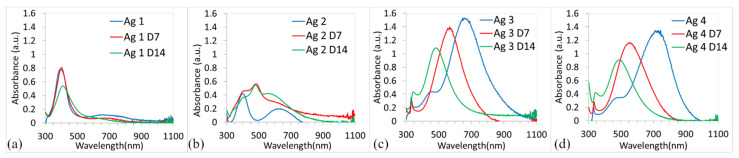
The stability of AgNPs with time for sample Ag 1 (**a**); sample Ag 2 (**b**); sample Ag 3 (**c**) and sample Ag 4 (**d**). The stability of AgNPs with time.

**Figure 3:**
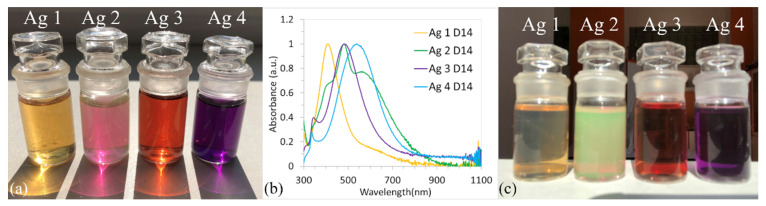
Color of AgNPs colloids on 14th day—view in transmitted light (**a**); absorption spectra of AgNPs colloids on 14th day (**b**); color of AgNPs colloids on 14th day—view in reflected light (**c**).

The stability of the colloidal solutions was analyzed over a period of one month. It is well known that silver nanoparticles are light-sensitive. Since all solutions were stored in the light and at room temperature, some changes in the color of the solution and, subsequently, in the SPR bands were observed. The UV-vis absorption spectra for all samples measured on the 1st, 7th, and 14th days are in Figure 2. The color of solutions after two weeks is shown in Figure 3a.

The absorption spectra of Ag 1 Figure 2a did not change significantly. No changes were observed on the 7th day; on the 14th day, a decrease in ABS_max_ from 0.8 to 0.5 was recorded. The presence of a single and symmetric absorption peak even after two weeks indicates that the shape of synthesized spherical nanoparticles did not change [26]. It can be assumed that synthesized nanoparticles have good stability, which also confirmed the unchanged color of the Ag 1 solution on the 14th day Figure 3a.

A different behavior showed samples Ag 3 and Ag 4 Figure 2c,d. The substantial blue shift of SPR bands and the decrease in ABS_max_ can be observed with time, whereas the shape of the curves did not change significantly. However, significant changes (compared to day 0, Figure 1a) were recorded in the colors of the solutions Figure 3a, where the color of samples Ag 3 and 4 changed from purple to red and from blue to purple, respectively. After this time, the color of the solutions stabilized and did not change even after a month (data not shown).

There are many scientific works that have confirmed that the change of color of a colloidal AgNPs solution is highly dependent on the size and shape of the nanoparticles. The etching of triangular nanoparticles in the presence of H_2_O_2_ can be the main reason. This phenomenon was studied by many authors [7,15,16]. Etching can result in dramatic changes in the SPR band shape and position. The truncation and size reduction of the AgNPs induced by the H_2_O_2_ etching results in the blue-shift of their optical properties from near-infrared to visible ranges; such behavior was also observed in works [7,27,28,29].

The significant blue shift from 720 nm to 540 nm of ABS_max_ occurred simultaneously with the decrease of ABS_max_ from 1.4 to 1.1 for sample Ag 3 Figure 2c. Similar behavior was observed with the sample Ag 4, where the blue shift from 660 nm to 480 nm and the decrease in ABS_max_ from 1.5 to 1 was observed Figure 2d. We assume that the slow surface oxidation of AgNPs or the formation of other shapes in samples Ag 3 and 4 occurred, which led to color change.

The most dramatic change in SPR bands was recorded in the Ag 2 sample Figure 2b. The SPR band dramatically changed during the experiment and not just the value of ABS_max_ and *λ*_max_, but also the shape of the SPR band changed. Based on the presence of the three peaks it is possible to hypothesize that either nanoparticles could be polydisperse (a mix of small and large nanoparticles with different shapes) or that non-symmetrical nanoparticles are presented in solution.

Figure 3b shows the comparison of normalized UV-vis absorption spectra of all samples on the 14th day. Compared to Figure 1b, the significant shift of ABS_max_ to a lower wavelength is only evident for samples Ag 3 and Ag 4.

A very interesting phenomenon can be observed in the case of the Ag 2 sample Figure 3c. The sample is dichroic, it shows different colors when viewed from different directions. The reason is different absorption coefficients for a light polarized in different directions. When viewed in reflected light, the solution is green in color, whereas when viewed in transmitted light, the solution appears pink Figure 3a. No such change was observed for the other three solutions Figure 3c. We hypothesized that this phenomenon is caused by a unique combination of sizes and shapes of nanoparticles. Similar optical properties of colloidal AgNPs solutions have been observed and studied by other authors [5,6,30]; based on their conclusions, the formation of the dichroic solution is due to the polydispersity of the nanoparticles (large and non-symmetrical particles).

Based on the UV-vis spectrum of the Ag 2 dichroic sample, we can assume the presence of polydisperse nanoparticles in the solution, which is consistent with the findings of other authors. Dekker et al. studied the dichroic solutions Ag and Au and showed that polydisperse asymmetric nanoparticles were present in all of them [5]. The dichroic effect was observed in AuNPs colloids by Koolo et al. and Jakhmola, and both found asymmetric nanoparticles in dichroic colloids [31,32,33]. This leads to the conclusion that the polydispersity and asymmetric shape of the nanoparticles is the main reason for the dichroic behavior. We, therefore, assume that the originally formed triangular nanoparticles (Ag 2 sample) changed to asymmetric (by etching, shortening), which in the end caused a dichroic effect. However, we also assume that etching caused the increase in Ag^+^ ions in the solution, which led to the formation of small nanoparticles in the range of up to 5–10 nm and as the stabilization agents are already presented in the solution, these nanoparticles are stabilized and no further growth occurs. However, this hypothesis about the formation of small irregular nanoparticles can only be confirmed by TEM/SEM analysis.

### 3.3. Electron Microscopy Analysis

**Figure 4:**
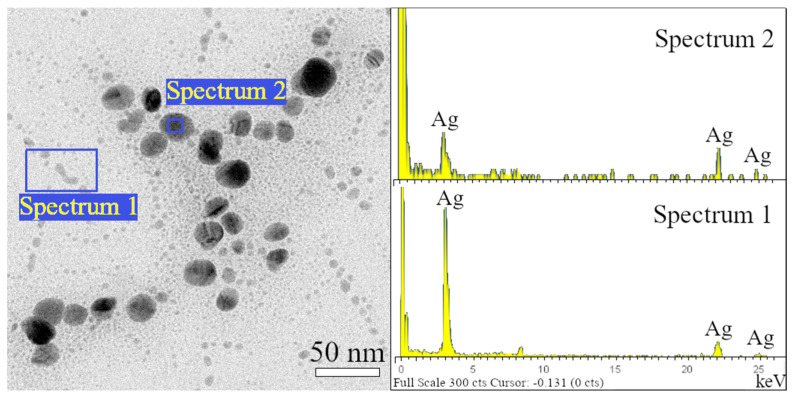
The energy dispersive *X*-ray spectroscopy of both size categories of AgNPs.

To confirm the presence of silver several techniques, such as Energy Dispersive *X*-ray Spectroscopy, diffraction analysis, and a digital method for lattice fringe spacing measurements in HRTEM were used. Analyzes were performed on all samples (for all sizes and shapes of nanoparticles). Figure 4 shows the results of energy dispersive *X*-ray spectroscopy of sample Ag 1; it is evident that a strong silver signal in both size categories (small and large nanoparticles) is presented. Such a strong silver signal was observed in all samples (data not shown).

**Figure 5:**
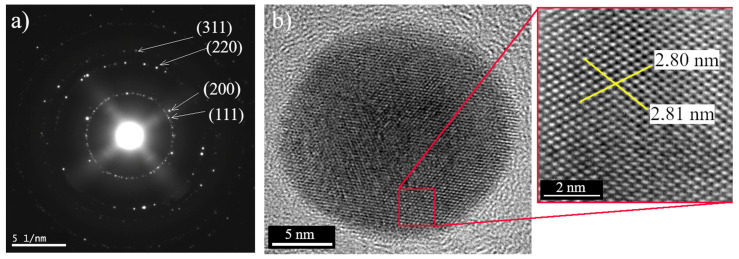
The electron diffraction of AgNPs (**a**); AgNPs structural lattice and lattice fringes of the synthesized AgNPs (**b**).

Figure 5 shows the electron diffraction pattern of the selected area of sample Ag 1 and a high-resolution transmission electron micrograph of an isolated nanoparticle with a size of ~19 nm. Figure 5a shows the characteristic reflections of face-centered cubic crystalline silver. Bright circular rings assigned to (111), (200), (220), and (311) are well observable in the selected area [33]. Based on diffraction results, we can also conclude that the synthesized nanoparticles formed as single crystals. The HR-TEM image Figure 5b shows the lattice fringe with 0.254 nm (2.80 nm for 11 atoms), which also confirms the crystalline nature of the synthesized AgNPs. The same results were also obtained for samples Ag 2, 3, and 4 (data not shown).

**Figure 6:**
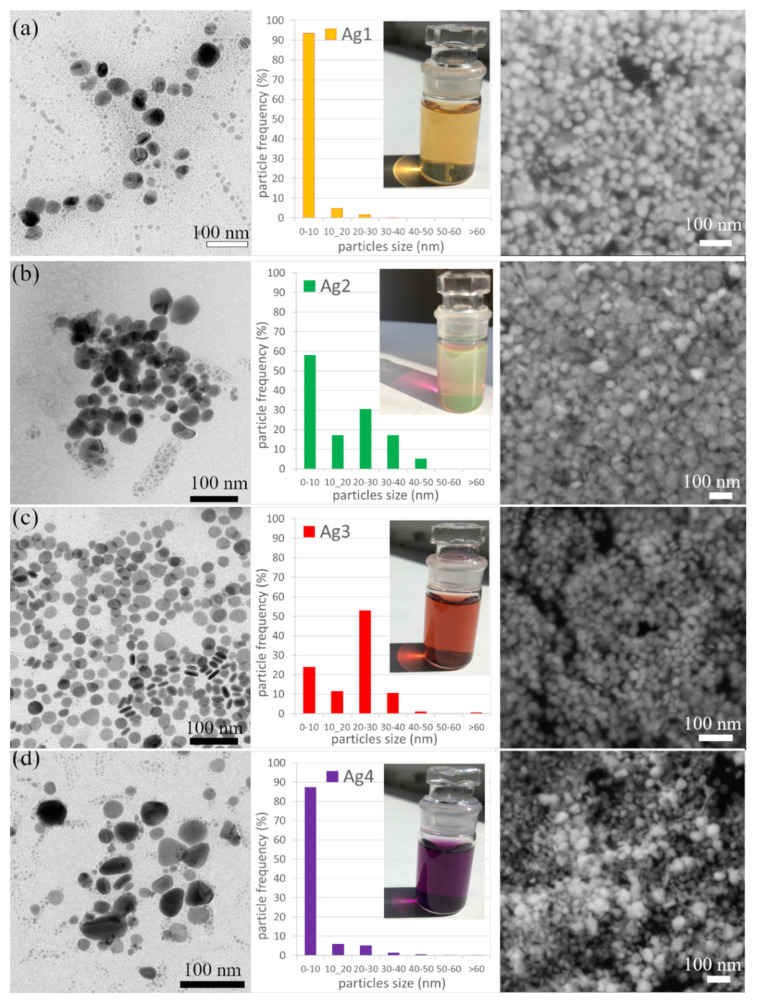
TEM images, size distribution histograms and SEM images of samples Ag 1 (**a**); Ag 2 (**b**); Ag 3 (**c**); and Ag 4 (**d**) on the 14th day of the experiment.

**Figure 7:**
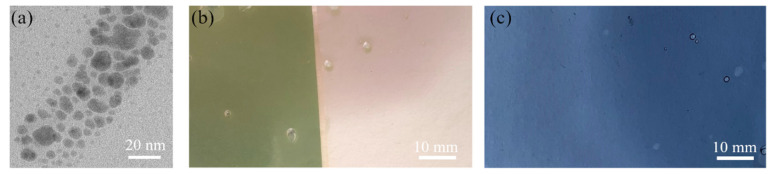
TEM image of small and irregular AgNPs, and AgNPs of quantum dots size (**a**); the thin layer of polymer composite with incorporated dichroic AgNPs (from left to right: reflected and transmitted light) (**b**); and triangular nanoparticles incorporated into PVA matrix (**c**).

The TEM images, size distribution histograms, and SEM micrographs of Ag 1, Ag 2, Ag 3, and Ag 4 samples (the 14th day) are shown in Figure 6. The TEM images show that all samples contain small spherical nanoparticles with a diameter < 10 nm, however, there are also some substantial differences between samples:

1. The sample Ag 1 contains 94% of nanoparticles with a mean diameter up to 10 nm. The max particle diameter was 30 nm (6%). The TEM and SEM images confirmed that all nanoparticles are spherical or near-spherical.
2. The sample Ag 2 contains 58% of nanoparticles with a mean diameter up to 10 nm, where even the nanoparticles in the size of quantum dots (<5 nm, 40%) were observed Figure 7a. Only 0.6% of the particles were >50 nm in diameter (max diameter 57 nm). The TEM and SEM images confirmed that nanoparticles are polydisperse, near-spherical, and irregular. Nanoparticles with a mean diameter up to 10 nm are non-symmetrical (58%) Figure 7a. We assume that such shape distribution (irregularity and the presence of small nanoparticles in the range of up to 5 nm) caused a dichroic effect Figure 6b. There are several works dealing with the explanation of the dichroic effect. In his work, Caseri analyzed several works from which, it is clear, that there are discrepancies in the explanation of the dichroic effect, and there are several hypotheses about what this effect (in the case of colloidal solutions) causes [30]. Most authors agree that anisotropic metal particles are responsible for the dichroic effect. However, other authors argue that the particles must be arranged in a particular system in order to achieve this effect. Others argue that dichroism does not come from anisotropic crystal modification of metal particles but rather from rod-shaped metal particles that are able to arrange in a certain spatial arrangement. Based on our observations and TEM photographs, we conclude that anisotropic nanoparticles measuring about 5 nm are the main reason for the dichroic effect, but at the same time, larger nanoparticles of irregular shapes (~30–40 nm) must be present in the solution. No other regular shapes were observed in our dichroic solution, e.g., rod-like or triangles. These are present in combination with quasi-spherical nanoparticles in Ag 3 and Ag 4 solutions, but these solutions are not dichroic.
3. The sample Ag 3 contains only 24% of nanoparticles with a mean diameter up to 10 nm and a larger number of nanoparticles with a diameter up to 30 nm (64%). There were also observed particles with a mean diameter >60 nm (0.4%). The TEM and SEM images confirmed that the mix of spherical and rod-like nanoparticles is present (the ratio spherical:rodlike = 3:1).
4. The sample Ag 4 contains the largest amount (87%) of nanoparticles with a mean diameter up to 10 nm and only 13% of larger nanoparticles. The TEM and SEM images confirmed that small nanoparticles are all spherical, larger nanoparticles have mostly irregular, quasi-spherical shapes, and a small number of triangular nanoparticles were also observed.

Figure 7b shows the thin layer of polymer composite prepared by the incorporation of dichroic AgNPs (sample Ag 2) into the PVA matrix by the “ex-situ” method. Silver nanoparticles did not lose their dichroic properties when incorporated into the polymer matrix. It is obvious that the PVA-AgNPs composite is pink in transmitted light, but in the reflected light it is green. Figure 7c shows the thin layer of polymer composite prepared by the incorporation of triangular silver nanoparticles (sample Ag 4). As in the case of a colloidal solution (sample Ag 4) Figure 1a, the triangular nanoparticles color the polymer matrix also blue. Sample Ag 1 and Ag 3 colored the PVA matrix in yellow and red, respectively (data not shown).

Dichroic materials can be widely used in science and art. The dichroic solution can be used for dichroic polymer and glass composite preparation. Dichroic filters produce light that is perceived by humans to be highly saturated in color. This property can be used in architectural and theatrical applications. Dichroic filters and glasses are currently produced by lamination (the material is evaporated in a vacuum and then applied to the surface in several layers), which is not the easiest and cheapest way to prepare. The use of chemically prepared nanoparticles in the production of dichroic materials could greatly facilitate and reduce the cost of current production methods. There is also the possibility of incorporating nanoparticles into polymers and biopolymers [6,30]. The polymers thus formed have a stable color, and since silver is known for its excellent antibacterial properties, it is likely that the polymer composites will also exhibit antibacterial properties.

## 4. Conclusions

A cheap, easy, and fast chemical method for the synthesis of AgNPs was used. We have successfully synthesized the AgNPs colloids with different shaped nanoparticles (spherical, triangular, rodlike). It was proven that the shape of nanoparticles and their stability depend on the reactant concentration. A higher concentration of reduction agents leads to the formation of rodlike and triangular nanoparticles, and lower to the formation of spherical nanoparticles. The etching of triangle particles to the quasi-spherical and irregular nanoparticles of different sizes was observed during the experiment. By changing a shape and size of AgNPs, the dichroic effect was achieved. The dichroic effect was displayed by color changes as light passed through the sample. The solution was pink in the transmitted light and green in the translucent light. Such simple preparation of dichroic nanoparticles and their incorporation into a polymer matrix can replace expensive technologies to produce dichroic materials.

The nanoparticles have retained their optical properties even after incorporation into the polymer matrix (PVA). We have successfully prepared a dichroic polymer composite (pink-green) by introducing dichroic AgNPs into the polymer. The incorporation of the triangular nanoparticles (sample Ag 4) created a blue-colored polymer composite and similarly the nanoparticles from the Ag 1 and Ag 3 samples yellow and red, respectively. Other dichroic color combinations can also be achieved by changing the conditions of synthesis. We have shown that the simple and relatively inexpensive method of preparing nanoparticles and polymer composites can be used for preparing materials with very attractive optical properties.

## Figures and Tables

**Table 1:** Reagents concentrations.

Reagent	Amount (mL)	Concentration
Silver nitrate	130	0.11 mM
Sodium citrate	11	30 mM
Polyvinylpyrrolidone	11	2% *w*/*w*
Hydrogen peroxide	0.36	30% *w*/*w*
Sodium borohydride	0.45; 0.84; 1; 1.5	0.1 M

## Data Availability

Not applicable.
